# Oligoarthritis Caused by *Borrelia bavariensis*, Austria, 2014

**DOI:** 10.3201/eid2106.141516

**Published:** 2015-06

**Authors:** Mateusz Markowicz, Stefan Ladstätter, Anna M. Schötta, Michael Reiter, Gerhard Pomberger, Gerold Stanek

**Affiliations:** Medical University of Vienna, Vienna, Austria (M. Markowicz, A.M. Schötta, M. Reiter, G. Stanek);; Donauspital, Vienna (S. Ladstätter, G. Pomberger)

**Keywords:** Lyme disease, Lyme borreliosis, *Borrelia bavariensis*, bacteria, arthritis, oligoarthritis, PCR, *Suggested citation for this article*: Markowicz M, Ladstätter S, Schötta AM, Reiter M, Pomberger G, Stanek G. Oligoarthritis caused by *Borrelia bavariensis*, Austria, 2014. Emerg Infect Dis. 2015 Jun [*date cited*]. http://dx.doi.org/10.3201/eid2106.141516

## Abstract

A case of Lyme oligoarthritis occurred in an 11-year-old boy in Vienna, Austria. DNA of *Borrelia bavariensis* was detected by PCR in 2 aspirates obtained from different joints. Complete recovery was achieved after a 4-week course with amoxicillin. Lyme arthritis must be considered in patients from Europe who have persisting joint effusions.

Lyme borreliosis is a tickborne disease caused by certain species of spirochetes of the *Borrelia burgdorferi* sensu lato (s.l.) complex. In Europe, several genospecies of *B. burgdorferi* s.l. cause the disease, whereas in North America, *B. burgdorferi* sensu stricto is the only agent of Lyme borreliosis. This difference causes variability in clinical manifestations (Figure). According to surveillance by the US Centers for Disease Control and Prevention, Lyme arthritis occurs in 30% of Lyme borreliosis patients in the United States (*1*), whereas in Europe, arthritis is reported in only 3%–7% of patients, as assessed in a few epidemiologic studies (*2**,**3*). Direct comparison of the frequencies of clinical manifestations is difficult because of possible differences in case definitions.

**Figure:**
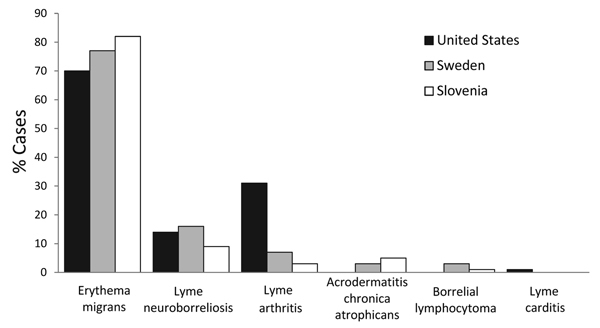
Comparison of frequency of clinical manifestations in Lyme borreliosis cases between the United States and 2 countries in Europe. Data from the United States are based on 154,405 patients identified during 2001–2010 by Centers for Disease Control and Prevention surveillance (*1*). Cases in Europe are represented by data from southern Sweden (1,471 patients, 1992–1993) (*2*) and Slovenia (1,020 patients, 2000) (*3*). The category Lyme neuroborreliosis includes all neurologic manifestations, such as radiculoneuropathy, facial palsy, and meningitis or encephalitis. Some patients had >1 manifestation.

In most cases, diagnosis of Lyme arthritis is made on the basis of the clinical picture supported by serologic testing. PCR testing of synovial fluid or synovial tissue samples is the most reliable method for direct identification of the pathogen (*4*). Cultivation of the pathogen from these materials is difficult, and recovery has been reported only anecdotally.

Lyme arthritis usually affects 1 or several large joints, most commonly the knee (*3*). Several studies, mostly of serologic testing and clinical picture, have shown different patterns of joint involvement in children (*5*). Therefore, it is difficult to distinguish Lyme arthritis from other forms of arthritic diseases, particularly juvenile idiopathic arthritis, on the basis of clinical signs and symptoms. Both diseases may present with oligoarticular involvement with symmetrically or unilaterally occurring joint effusions. We report a case of Lyme oligoarthritis in an 11-year-old boy from Vienna, Austria.

## The Study

A signed consent form was obtained from the mother of the patient. The patient had reported recurrent joint pain, most prevalent in his left knee, since he was 6 years old. In December 2012, when he was 10 years old, his left knee became swollen, and he was treated locally with nonsteroidal antiinflammatory drugs.

In February 2014, the patient had effusions of both knees and the left ankle. The first joint aspiration of the left knee was performed in February 2014, but the patient was discharged without a diagnosis. Soon after that, he became febrile (temperature 39°C) and was referred to another hospital because of persistent effusions of all 3 joints. Clinical investigation revealed swelling of both knees, which were not warm or red, and a swollen, hot, red left ankle.

A routine blood test showed a normal leukocyte count, elevated C-reactive protein levels (79.4 mg/L, positive threshold 5 mg/L), and elevated erythrocyte sedimentation rates (85 mm/h, reference <7 mm/h, and 109 mm/2 h, reference <12 mm/2 h). Tests for rheumatoid factor and other autoantibodies (antinuclear antibodies, double-stranded DNA, proteinase 3, myeloperoxidase antibodies) showed negative results. The patient underwent needle aspiration of all 3 joints under general anesthetic to obtain synovial fluid: 18 mL from the right knee, 60 mL from the left knee, and 6 mL from the left ankle. The patient was given a preliminary diagnosis of juvenile idiopathic arthritis and treated with nonsteroidal antiinflammatory drugs. A few days later, another aspiration of the left knee was performed, followed by an intraarticular injection of steroids.

Infection with pathogens associated with reactive arthritis was ruled out by negative serologic test results for *Chlamydia* spp. (IgG and IgA enzyme immunoassays), *Mycoplasma pneumoniae* (IgG and IgA enzyme immunoassays), *Salmonella* spp. (agglutination assay), and *Yersinia* spp. (agglutination assay). Antibodies against *B. burgdorferi* s.l. were detected by *Borrelia* ELISA (Medac, Hamburg, Germany); IgG levels were highly elevated (IgG ELISA >200 AU/mL, cutoff 10.8 AU/mL; IgM ELISA results were negative). Test results obtained by using the Anti-*Borrelia* Euroline Westernblot (Euroimmun, Lübeck, Germany) were positive for IgG with strong band intensities for VlsE, p83, p39, p30, p21, p19, and p17 and weak band intensity for p25 (OspC). Cytologic test results for synovial fluid showed an inflammatory infiltrate with lymphocytes and segmented neutrophils. Culture for bacterial pathogens was negative.

Synovial fluid samples from all 3 joints were tested by using PCR. DNA was extracted by using the PeqGOLD Tissue DNA Mini Kit (Peqlab, Erlangen, Germany). Two TaqMan-based real-time PCR assays targeting the 16S rDNA gene (*6*) and the flagellin gene (*7*) were performed; primer and probe sequences are listed in Table 1. The DNA of an in-house *B. burgdorferi* s.l. strain was used as a positive control, and PCR-grade water was used as a negative control. To check for PCR inhibition, we used samples spiked with borrelial DNA in an extra well. In 2 of the 3 joint aspirates (left knee and left ankle), borrelial DNA was detected by both assays. For genotype identification, samples were subjected to a previously described nested PCR targeting the 5S-23S intergenic spacer region (*8*).

**Table 1:** Primer and probe sequences used in identification of *Borrelia bavariensis* in 11-year-old patient with oligoarthritis, Vienna, Austria, February 2014*

Primer or probe	Target gene	Sequence, 5′ → 3′	Reference
Primer			
16SF	16S rDNA	GCT GTA AAC GAT GCA CAC TTG GT	(*6*)
16SR	16S rDNA	GGC GGC ACA CTT AAC ACG TTA G	(*6*)
BorF	Flagellin	GAA TTA GCA GTT CAA TCA GG	(*7*)
BorR	Flagellin	TTC GTC TGT AAG TTG CTC TAT	(*7*)
rrf-rrl IGS F	5S–23S IGS	CTG CGA GTT CGC GGG AGA	(*8*)
rrf-rrl IGS R	5S–23S IGS	TCC TAG GCA TTC ACC ATA	(*8*)
B5S-23S_Fn	5S–23S IGS	GAG TTC GCG GGA GAG TAA G	(*8*)
B5S-23S_Rn	5S–23S IGS	TAG GCA TTC ACC ATA GAC TCT T	(*8*)
V1a	*ospA *	GGG AAT AGG TCT AAT ATT AGC	(*10*)
V1b	*ospA *	GGG GAT AGG TCT AAT ATT AGC	(*10*)
V3a	*ospA *	GCC TTA ATA GCA TGT AAG C	(*10*)
V3b	*ospA *	GCC TTA ATA GCA TGC AAG C	(*10*)
R1	*ospA *	CAT AAA TTC TCC TTA TTT TAA AGC	(*10*)
R37	*ospA*	CCT TAT TTT AAA GCG GC	(*10*)
Probe			
LBTM	16S rDNA gene	FAM–TTC GGT ACT AAC TTT TAG TTA A–TAMRA	(*6*)
BorTM	Flagellin gene	FAM–AAC GGC ACA TAT TCA GAT GCA GAC–TAMRA	(*7*)

*IGS, intergenic spacer.

Amplicons were purified by using the QIAquick PCR Purification Kit (QIAGEN, Hilden, Germany) and sent to MWG Eurofins (Munich, Germany) for bidirectional sequencing by using primers B5S-23S_Fn and B5S-23–S_Rn (*8*). Sequencing revealed the same *Borrelia* strain in both joints. When compared with known sequences by using BLAST (http://www.ncbi.nlm.nih.gov/blast), the sequences showed 100% identity with that of the PBi strain.

Because the PBi strain and all PBi-like strains are now known as a distinct genospecies within the *B. burgdorferi* s.l. complex, namely *B. bavariensis* (*9*), and because *B. bavariensis* strains are associated with OspA serotype 4, another PCR for identifying the OspA serotype was carried out. To determine the OspA type of the *Borrelia* spp. found in the joint aspirates, we performed another previously described nested PCR targeting the *ospA* gene (*10*), then sequenced and identified a virtual restriction fragment polymorphism by using CLC Main Workbench version 7.0 (CLC bio, Aarhus, Denmark). Because the obtained restriction fragment length polymorphism pattern matched OspA serotype 4, the presence of a *B. bavariensis* strain in both *Borrelia-*positive joints could be confirmed.

After the patient was treated with amoxicillin (500 mg 3×/d for 28 days) (*4*), all joint effusions resolved. At his 9-month follow-up visit, the patient did not report any symptoms. No erythema migrans was observed, and the patient’s mother reported only 1 tick bite for the patient when he was 2 years old.

## Conclusions

In Europe, Lyme arthritis can be caused by several genospecies of *B. burgdorferi* s.l. Published studies have most commonly identified *B. burgdorferi* sensu stricto in joints from Lyme arthritis patients in Europe (Table 2). *B. bavariensis*, the pathogen that caused the illness in the patient we describe, was formerly classified as *B. garinii* genospecies characterized by OspA serotype 4 (*9*). On the basis of multilocus sequence analysis of chromosomal housekeeping genes, this group was found to be genetically distinct from other *B. garinii* strains. Furthermore, the 2 genotypes differ in their hosts: *B. bavariensis* is a rodent-associated strain, whereas other *B. garinii* serotypes can be found in birds.

**Table 2:** Reports of Lyme arthritis and identified genospecies in patients in Europe*

Study	Year published	PCR target	Total no. cases	PCR-positive cases	*Borrelia* spp., no. (%) identified		
					*B. burgdorferi* sensu stricto	*B. afzelii*	*B. garinii*

Eiffert et al. (*11*)	1998	*ospA* gene	11	7	3 (43)	1 (14)	3 (43)
Vasiliu et al. (*12*)	1998	*ospA* gene	20	13	4 (31)	5 (38)	4 (31)
van der Heijden et al. (*13*)	1999	5S–23S IGS	4	3	3 (100)	0	0
Jaulhac et al. (*14*)	2000	Flagellin gene	12	10	9 (90)	0	1 (10)
Total			47	33	19 (58)	6 (18)	8 (24)

*IGS, intergenic spacer.

Most Lyme arthritis patients respond well to a single course of treatment with antimicrobial drugs, although in a small percentage of cases persistent synovitis can develop months or even years after treatment. For those patients whose synovial fluid PCR result is negative, intraarticular application of corticosteroids can be beneficial (*15*).

This case illustrates that Lyme arthritis must be taken into account in patients in Europe who have persisting joint effusions. Treatment with antimicrobial drugs is highly effective. We did not find any other report of cases in which the pathogen was detected in multiple joints by using a direct identification method. This case is further evidence for the systemic characteristics of Lyme borreliosis.
